# Development and Characterization of High-Absorption Microencapsulated Organic Propolis EPP-AF^®^ Extract (i-CAPs)

**DOI:** 10.3390/molecules28207128

**Published:** 2023-10-17

**Authors:** Andresa A. Berretta, Jéssica A. De Lima, Soraia I. Falcão, Ricardo Calhelha, Nathaly Alcazar Amorim, Isabella Salgado Gonçalves, Luana Gonçalves Zamarrenho, Hernane da Silva Barud, Jairo Kenupp Bastos, David De Jong, Miguel Vilas-Boas

**Affiliations:** 1 Department of Research, Development & Innovation, Apis Flora Indl. Coml. Ltd.a., Ribeirão Preto 14020-670, Brazil; jessica.lima@apisflora.com.br (J.A.D.L.); nathaly_alcazaramorim@hotmail.com (N.A.A.); luana.zamarrenho@apisflora.com.br (L.G.Z.); 2 Department of Pharmaceutical Sciences, Faculty of Pharmaceutical Sciences of Ribeirão Preto, University of São Paulo, Ribeirão Preto 14040-903, Brazil; jkbastos@usp.br; 3 Centro de Investigação de Montanha (CIMO), Instituto Politécnico de Bragança, Campus de Santa Apolónia, 5300-253 Bragança, Portugal; sfalcao@ipb.pt (S.I.F.); calhelha@ipb.pt (R.C.); mvboas@ipb.pt (M.V.-B.); 4 Laboratório Associado para a Sustentabilidade e Tecnologia em Regiões de Montanha (SusTEC), Instituto Politécnico de Bragança, Campus de Santa Apolónia, 5300-253 Bragança, Portugal; 5 Biopolymers and Biomaterials Group, University of Araraquara, UNIARA, Araraquara 14801-320, Brazil; isabella.salgado@hotmail.com (I.S.G.); hernane.barud@gmail.com (H.d.S.B.); 6 Department of Neuroscience and Behavior, Ribeirão Preto Medical School, University of São Paulo (FMRP/USP), Ribeirão Preto 14049-900, Brazil; 7 Genetics Department, Ribeirão Preto Medical School, University of São Paulo (FMRP/USP), Ribeirão Preto 14049-900, Brazil; ddjong@fmrp.usp.br

**Keywords:** microencapsulated propolis extract, antitumor, anti-inflammatory, antioxidant, antimicrobial, bioavailability, permeability

## Abstract

The demand for organic and functional food continues to increase yearly. Among the available functional foods, propolis is a bee product that has various beneficial properties, including antimicrobial, antioxidant, and anti-inflammatory activities. However, it generally is only available in ethanol solution, which has poor bioavailability, as it is relatively insoluble in water. The use of such ethanol extracts is often objectionable because of the alcohol content and because they have a strong and striking taste. Development of alternatives that can efficiently and safely increase solubility in water, and that meet organic production specifications, has been a challenge. To address these concerns, microcapsules were developed using spray-dryer technology from an emulsion based on EPP-AF^®^ propolis and gum arabic (i-CAPS). These propolis-loaded microcapsules were characterized using FT-IR, SEM, TGA, HPLC, and spectrophotometric techniques, along with determination of antimicrobial, antioxidant, antitumor, anti-inflammatory, and antihypercholesterolemic activities, as well as permeability in in vitro models. The production system resulted in microcapsules with a spherical shape and an encapsulation efficiency of 93.7 ± 0.7%. They had IC50s of 2.654 ± 0.062 and 7.342 ± 0.058 µg/mL by FRAP and DPPH antioxidant methods, respectively. The EPP-AF^®^ i-CAPS also had superior antimicrobial activity against Gram-positive bacteria. Antitumor activity was calculated based on the concentration that inhibited 50% of growth of AGS, Caco-2, and MCF-7 cell strains, giving results of 154.0 ± 1.0, 117 ± 1.0, and 271.0 ± 25 µg/mL, respectively. The microcapsule presentation reduced the permeation of cholesterol by 53.7%, demonstrating antihypercholesterolemic activity, and it improved the permeability of *p*-coumaric acid and artepillin C. The IC50 for NO production in RAW 264.7 cells was 59.0 ± 0.1 µg/mL. These findings demonstrate the potential of this new propolis product as a food and pharmaceutical ingredient, though additional studies are recommended to validate the safety of proposed dosages.

## 1. Introduction

The market for food supplements is experiencing significant growth due to increasing consumer interest in products that promote health and well-being. In 2020, the global dietary supplements market was valued at USD 140.3 billion. It is projected to reach USD 235.7 billion by 2028, with a compound annual growth rate of 6.9% [1]. This market expansion reflects the growing awareness and demand for supplements that offer nutritional support and potential health benefits.

Similarly, the organic food market has been witnessing remarkable growth worldwide. In 2020, the global organic food and beverage market value was estimated at USD 91.99 billion and is expected to reach USD 320.5 billion by 2027, with an annual growth rate of 16.3% [1]. Consumers are increasingly seeking organic products due to concerns about health, sustainability, and environmental impact.

Propolis is a bee product produced by *Apis mellifera* bees from plant parts and exudates that they collect and transform with their secretions. The chemical composition of propolis is variable according to the botanical sources, region of origin, and seasonality [2,3]. This affects its qualitative and quantitative chemical characteristics, resulting in many distinct propolis types, as is common with phytomedicines [4]. To circumvent this variability, a green propolis standardized extract (EPP-AF^®^) with reproducible batch-to-batch chemical profile and antimicrobial, wound-healing, anti-inflammatory, and antioxidant activities has been developed [5,6,7].

As the materials that bees use to produce propolis are collected by them from diverse botanical sources, the potential for contamination with pollutants varies depending on the region and specific plants involved [8]. Poplar propolis, for example, is susceptible to heavy metal accumulation and pesticide residues due to the characteristics of this type of tree and the environments where it grows [9]. Organic propolis extract, which is obtained from bees that are minimally exposed to pesticides and environmental pollutants, is significantly less contaminated [6,10]. On the international market, green propolis is particularly renowned and valued, not only because of its therapeutic benefits but also because of the low content of heavy metals, environmental pollutants, and antibiotic residues. The Africanized bees used in Brazilian apiculture are very resistant to diseases and parasites and beekeepers do not treat their colonies with antibiotics or acaricides [6,10,11], which favors organic propolis production and certification. Brazil is fortunate to be the exclusive producer of green propolis for the export market [6]. By 2017, Brazil exported approximately 120 tons of crude propolis yearly; subsequently, exports increased 30% as a consequence of the demand caused by the COVID-19 pandemic [11].

Propolis in its raw, crushed form is available in some markets; however, given its complex composition that involves waxes, resins, and non-absorbable materials, propolis cannot be consumed in this form and must be purified by an extraction process using suitable solvents to remove inert materials while preserving the polyphenolic fraction. In addition to its low solubility in water, propolis is poorly bioavailable, which has limited its use [12,13,14].

Propolis, known for its potential health benefits, has gained recognition in the functional food and nutraceutical industries [9,15]. However, the limited bioavailability and absorption of bioactive propolis compounds makes it difficult to fully harness its therapeutic potential. Given market demands and current propolis extract limitations, the development of new technologies that can provide organic food supplements based on propolis extract that have an improved aqueous absorption profile would be highly beneficial.

Microencapsulation, as a novel option, addresses this challenge by encapsulating propolis extract in protective microcapsules. This technique not only enhances the stability and solubility of the bioactive compounds but also allows for controlled release, improving their bioavailability and targeted delivery [14,16]. Using organic and sustainable materials in the microencapsulation process aligns with the increasing consumer demand for products that are natural and environmentally friendly.

Microencapsulation of propolis-based organic food supplements can offer numerous advantages. The utilization of carriers helps protect and preserve organic compounds during digestion, facilitating efficient absorption in the gastrointestinal tract [13,14,17]. Consequently, the bioavailability of bioactive propolis components can potentially be enhanced, maximizing their health benefits. We examined how microencapsulation of green propolis organic extract EPP-AF^®^ affects its biological and safety properties, as well as permeation behavior, in order to determine if it significantly and safely improves bioavailability.

## 2. Results

### 2.1. Development and Characterization

#### 2.1.1. Chemical Characterization 

**Figure 1:**
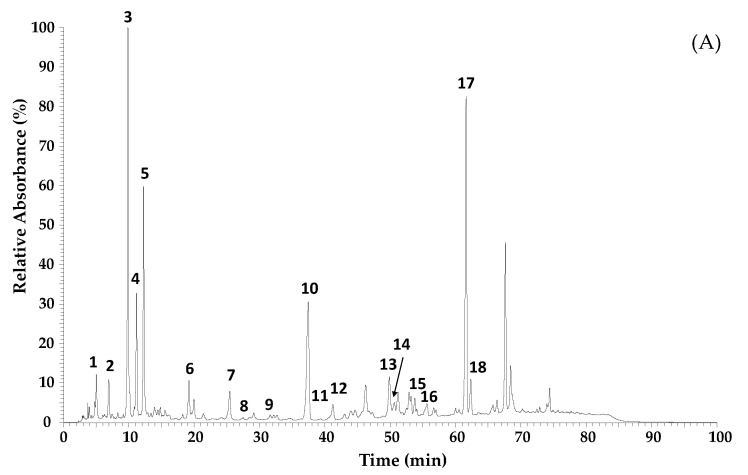

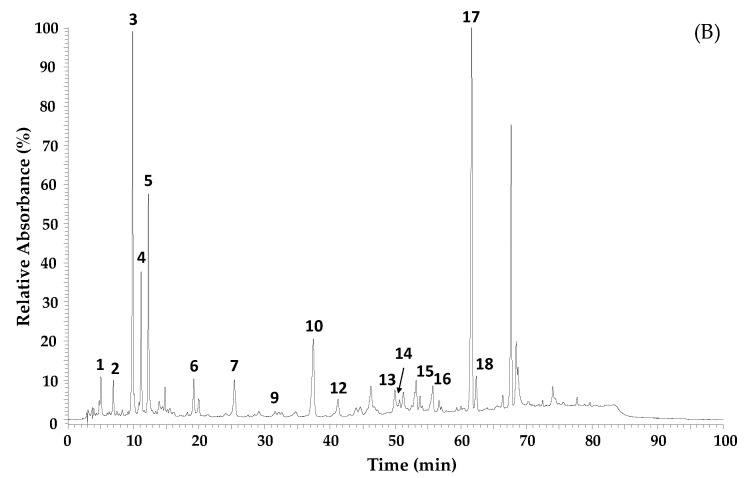
Chromatographic profiles obtained by LC/DAD/ESI-MS^n^ of organic green propolis standardized extract EPP-AF^®^ (**A**) and organic EPP-AF^®^ extract-loaded microcapsules (EPP-AF^®^ i-CAPS) (**B**).

Organic EPP-AF^®^ liquid extract at 11% *w*/*v* (propolis dry matter) was used to produce the EPP-AF^®^ i-CAPs; they presented 7.42 ± 0.14 mg/g and 13.83 ± 0.18 mg/g of total flavonoids and total phenolics, respectively. The microcapsules were found to contain 24.65 ± 0.64 mg/g of total flavonoids expressed as quercetin and 43.99 ± 1.17 mg/g of phenolic compounds, expressed as gallic acid. Figure 1A shows the chemical fingerprint of the EPP-AF^®^ and Figure 1B the organic propolis-loaded microcapsules, demonstrating that the key polyphenols were maintained, i.e., the encapsulation process did not affect the general profile of green propolis (EPP-AF^®^) extract (Table 1). Nevertheless, some minor changes occurred in the relation between the maximum intensity for peak 3 (*p*-coumaric acid) and peaks 4, 17, and 10, with an increase in the first two, dicaffeoylquinic acid and artepillin C, and a decrease in the relative intensity for the latter, drupanin (Figure 1B).

The physicochemical stability of EPP-AF^®^-loaded microcapsules was evaluated by the HPLC/DAD method after 36 months of shelf-life (samples stored in glass flasks protected from light at 25 °C). The compounds monitored for this purpose, *p*-coumaric acid, artepillin C, and total bioactive components, compared to the corresponding external standard, decreased 2.71, 14.22, and 7.79%, respectively (Table 2).

Statistical significance was assessed by two-way analysis of variance (ANOVA), followed by a Tukey multiple comparisons test to evaluate the differences. Significance was accepted with *p* less than 0.05 using the statistical program GraphPad Prism—version 6.0.

#### 2.1.2. Microencapsulation Efficiency (%ME)

The %ME was calculated based on the difference between the total phenolic compounds (such as gallic acid) present inside and outside the microcapsules. This showed the percentage of total phenolics from propolis extract effectively encapsulated with this system (93.68 ± 0.7%).

#### 2.1.3. Morphology—Microscopic and Macroscopic Aspects

**Figure 2:**
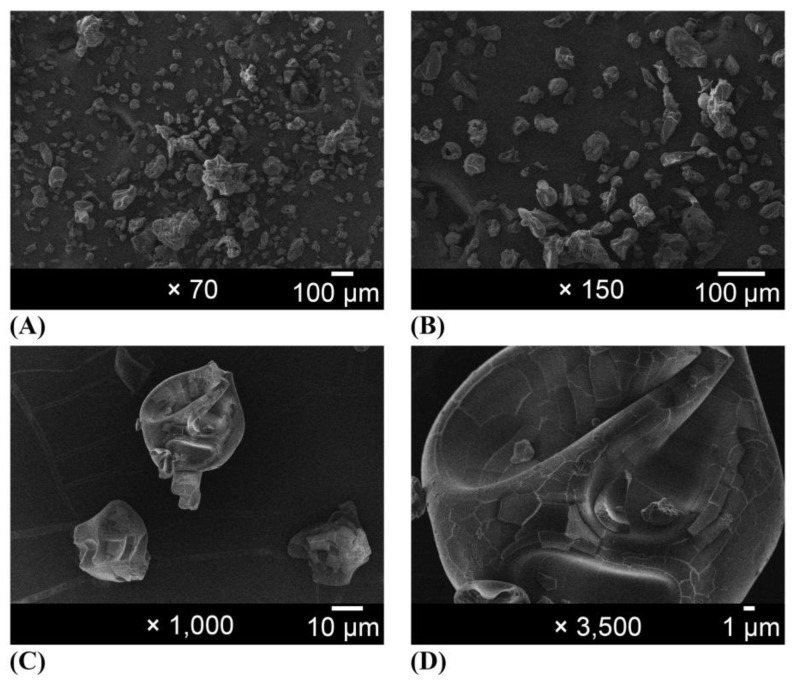
Photomicrographs of gum arabic magnified (**A**) 70×, (**B**) 150×, (**C**) 1000×, and (**D**) 3500×.

**Figure 3:**
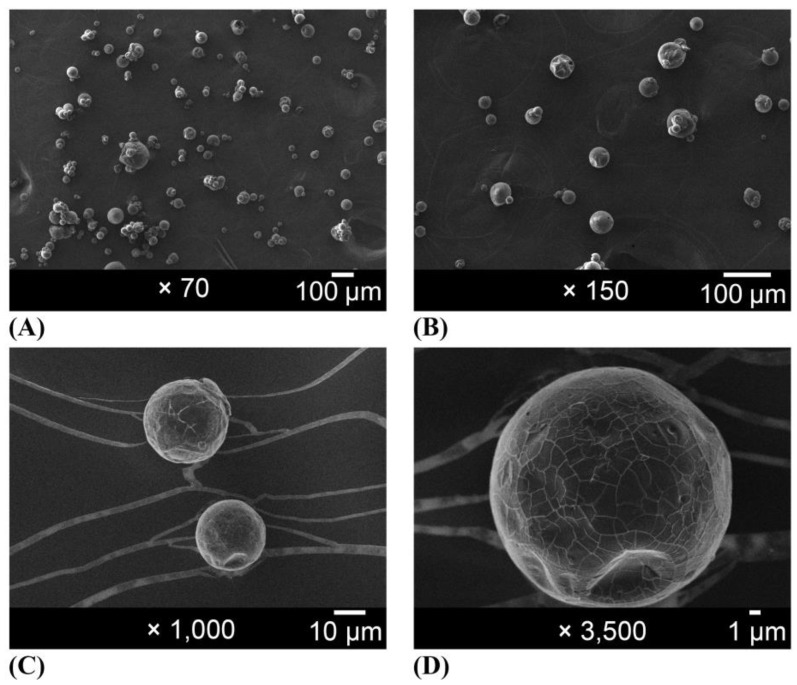
Photomicrographs of organic propolis standardized extract-loaded microcapsules (EPP-AF^®^ i-CAPs) magnified (**A**) 70×, (**B**) 150×, (**C**) 1000×, and (**D**) 3500×.

**Figure 4:**
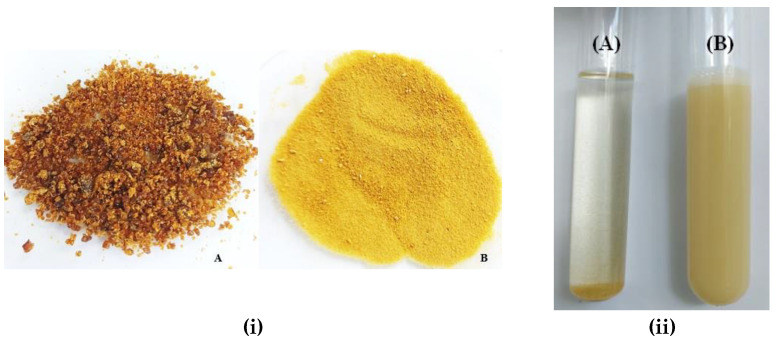
Visual aspect of lyophilized EPP-AF^®^ propolis formulation (**i.A**) and microencapsulated EPP-AF^®^ extract (**i.B**) and of the dispersion of EPP-AF^®^ extract (**ii.A**) and (**ii.B**) EPP-AF^®^ i-CAPS (0.5% *w*/*v* of propolis dry matter) in water.

Scanning electron microscopy images of the organic gum arabic and the organic EPP-AF^®^ propolis extract microcapsules are given in Figure 2 and Figure 3. For the macroscopic evaluation, the visual aspects of each powder alone and powder dispersed in purified water at 0.5% *w*/*w* of propolis dry matter were photographed (Figure 4). Before encapsulation, the gum arabic presented a varied to undefined morphology, with a heterogeneous particle size (Figure 2). The microcapsules were mostly spherical in shape and well defined (Figure 3) with an average size of 20 µm.

#### 2.1.4. FT-IR and TG/DTG/DSC Characterization

**Figure 5:**
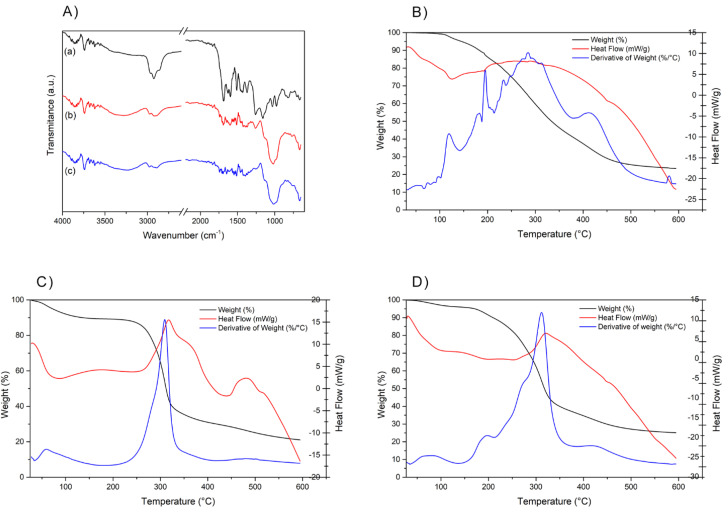
(**A**) Fourier transform infrared (FIT-IR) spectroscopy profile of propolis EPP-AF^®^ extract: control (a), gum arabic (b), and EPP-AF^®^-loaded microcapsules obtained-EPP-AF^®^ i-CAPs (c); thermal analysis profiles of (**B**) propolis EPP-AF^®^ extract, (**C**) gum arabic, and (**D**) EPP-AF^®^-loaded microcapsules.

Based on vibrational spectroscopy in the infrared region (Figure 5A) and analysis of the TG/DTG/DSC curves (Figure 5B–D), it can be observed that the encapsulation process of propolis with gum arabic results in structure and behavior of the microcapsules close to those of gum arabic. The FT-IR bands found for the propolis extract (Figure 5A-a) were around 1700 cm^−1^, 2920 cm^−1^, and in the region of 600 to 1160 cm^−1^, which correspond to the carboxyl group (COOH) present in organic compounds, CH stretching, OH bending, and C-O stretching, respectively. In the spectrum of gum arabic (Figure 5A-b), characteristic bands were found in the regions of 3200 to 3400 cm^−1^, 2900 cm^−1^, and 1600 cm^−1^, corresponding to O-H stretching, C-H stretching, and C=C stretching, respectively. Bands around 1700 cm^−1^ are also observed, indicating a COOH- group, while a band at 1400 cm^−1^ corresponds to O-H bending, and one around 1250 cm^−1^ represents C=O stretching [13].

In the vibrational spectrum of propolis microcapsules (Figure 5A-c), the characteristic bands of the gum arabic overlap the propolis extract bands. The characteristic band of propolis at around 1700 cm^−1^ appears reduced and shifted in the microcapsule spectra, suggesting successful microencapsulation. Smaller peaks would correspond to the chemical structural modifications mainly suffered by the aromatic rings [21]. This has been previously reported by other authors in other procedures using ethanolic-based encapsulation materials [13,22,23]. On the other hand, the microcapsule spectra show a broadening of the bands around 3500 cm^−1^, which corresponds to O-H stretching vibrations and may indicate interactions between propolis and gum arabic.

The thermogravimetry (TG/DTG) and differential scanning calorimetry (DSC) curves are shown in Figure 5B–D. The freeze-dried EPP-AF^®^ (Figure 5B) exhibited typical thermal behavior with a mass loss event (65%) starting at 150 °C and ending at 450 °C, as indicated by the DTG curve.

The gum arabic exhibited an initial thermal event with a mass loss of less than 10% between 100 and 150 °C, based on the TG/DTG curve, and an endothermic peak near 100 °C, indicated by the DSC curve (Figure 5C). This can be attributed to evaporation of surface water. A second event with a mass loss of approximately 50% occurring in the temperature range of 200 to 400 °C corresponds to decomposition of the polysaccharide, which is further evidenced by the exothermic peak in the DSC curve.

The propolis-loaded microcapsules exhibited intermediate thermal behavior between gum arabic and propolis, resembling the profile of gum arabic alone (Figure 5D). Similar to the gum arabic, the microcapsules showed a first mass loss event (less than 10%) between 50 and 150 °C and another mass loss of approximately 50% in the temperature range of 200 to 350 °C, demonstrated by the TG/DTG curve. The DSC curve shows an endothermic peak between 50 and 250 °C and an exothermic peak at around 350 °C, confirming degradation of the compound.

The mass loss was less in the encapsulation process when compared to the lyophilized sample. This demonstrates improvement in the thermal protection of propolis due to encapsulation.

#### 2.1.5. Release Study

**Figure 6:**
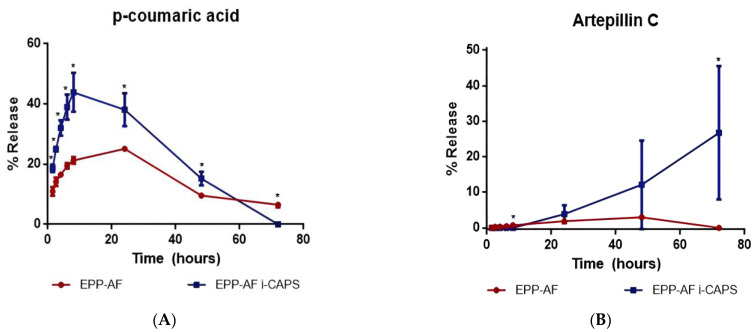
Delivery of the two biomarkers selected for this study, *p*-coumaric acid (**A**) and artepillin C (**B**), from EPP-AF^®^ extract and EPP-AF^®^-loaded microcapsules (*n* = 3, mean ± standard deviation). * Significantly different (*p* < 0.05).

The release of the *p*-coumaric acid biomarker (Figure 6A) occurred predominantly in the initial hours, likely due to its hydrophilic nature. The microcapsule formulation exhibited higher release of this biomarker in comparison with the control sample, indicating its efficacy in facilitating the release of *p*-coumaric acid, probably enhancing its permeation. This suggests that hydrophilic compounds have increased bioavailability in this microcapsule system. The release percentage exceeded 40% within the first eight hours. After 24 h, the release profile suggested that the biomarker begins to degrade within the receptor medium, although it continued to be gradually released in smaller quantities.

The release profile of artepillin C (Figure 6B) illustrates that, owing to its higher affinity for the microcapsule compared to the water present in the receptor medium, its release begins after a certain delay. The microcapsule enables a more substantial release of the active compound compared to the control, sustaining the release of artepillin C over extended periods. These findings suggest that the microcapsule system enhances the bioavailability not only of *p*-coumaric acid but also of artepillin C, which is highly advantageous for efficient use of propolis. Considering that approximately 30% of the active compound was released within 72 h, it can be inferred that sustained release persists for a more extended period, ensuring the permeation of this crucial biomarker in the relevant receptor medium.

The propolis microcapsule had a significantly higher release of the active compounds compared to the control lyophilized propolis extract. The statistical analysis included a *t*-test to compare the means and an F-test to compare the variances. The release profiles of the microcapsule and control were significantly different (*p* < 0.05).

The microcapsules gave an efficient and continuous release of the markers, with greater quantities released gradually over time. The release kinetics followed a zero-order model, wherein the release of the active compounds occurs as a function of time.

### 2.2. Biological Properties

#### 2.2.1. Antioxidant Activity

The antioxidant activity of EPP-AF^®^ and of EPP-AF^®^-loaded microcapsules was assessed using the FRAP and DPPH methods (Table 3). The propolis extract exhibited an antioxidant activity of 2.873 ± 0.045 µmol Fe(II)/mg propolis dry matter. In comparison, the propolis-loaded microcapsules had an antioxidant activity of 2.654 ± 0.062 µmol Fe(II)/mg propolis dry matter.

In the DPPH method, the antioxidant activity is expressed as IC50 values, which indicate the concentration required to scavenge 50% of the DPPH radicals. The propolis extract gave an IC50 value of 6.500 ± 0.062 µg propolis dry matter/mL, while the propolis-loaded microcapsules had an IC50 value of 7.342 ± 0.058 µg propolis dry matter/mL.

There was a significant difference in the antioxidant activity between the propolis extract and the microcapsules, as determined by both the FRAP and DPPH methods (*t*-test; *p* < 0.05; Table 3). The propolis extract had a higher antioxidant activity compared to the propolis-loaded microcapsules.

#### 2.2.2. Antimicrobial Activity

Table 4 and Table 5 present the antimicrobial data based on the minimum bactericidal concentration (MBC). Both the propolis extract and propolis-loaded microcapsules were diluted in a 40% *v*/*v* hydroethanolic solution (Table 4) and directly dispersed in Mueller–Hinton broth medium (Table 5). The propolis-loaded microcapsules were completely ruptured in a 40% *v*/*v* hydroethanolic solution. The results for the propolis extract and propolis-loaded microcapsules were found to be equivalent (as propolis dry matter), 3.44 mg/mL for *Staphyloccocus aureus* and 27.50 mg/mL for *Escherichia coli*, confirming that the active components of propolis were maintained and the procedures employed for the microencapsulation step were effective in preserving the antimicrobial activity of EPP-AF^®^ and that the resolubilization of lyophilized propolis and the microcapsules was efficient.

The objective of this protocol was to compare the biological characteristics of EPP-AF^®^ i-CAPs with the control (lyophilized EPP-AF^®^). The procedure involved direct dispersion of both samples in Mueller–Hinton broth medium. The results obtained with the broth microdilution method are presented in Table 5; the microcapsules exhibited higher antimicrobial activity against all the microorganisms compared to the propolis extract used as a control. A lower value in this method indicates greater potency of the sample.

The results for EPP-AF^®^ i-CAPs against the microorganisms *S. aureus, methicillin-resistant S. aureus (MRSA),* and *Staphylococcus epidermidis* were 1.72 ± 0.0, 5.73 ± 1.98, and 6.88 ± 0.0 mg/mL, respectively. In contrast, the control lyophilized EPP-AF^®^ presented values of 55.0 ± 0.0, 110.0 ± 0.0, and 55.0 ± 0.0 mg/mL for the same sequence of microorganisms.

Regarding the Gram-negative bacteria, although the results for EPP-AF^®^ i-CAPs were superior to those of the control, they were clearly less potent than against the Gram-positive microorganisms. The MBC values for *E. coli*, *Klebsiella pneumoniae*, and *Pseudomonas aeruginosa* with EPP-AF^®^ i-CAPs were 55.0 ± 0.0, 27.50 ± 0.0, and 36.67 ± 15.88 mg/mL, respectively. In comparison, the MBC values for EPP-AF^®^ against the same bacteria were 110.0 ± 0.0, 91.67 ± 31.75, and 91.67 ± 31.75 mg/mL, respectively.

The results were expressed as mean ± standard deviation obtained in three independent experiments. Statistical significance was assessed by two-way analysis of variance (ANOVA) followed by Sidak’s multiple comparisons test to evaluate statistical differences. Significance was accepted with *p* less than 0.05 using the statistical program GraphPad Prism—version 6.0.

#### 2.2.3. Cytotoxicity, Anti-Inflammatory, and Antihypocholesterolemia Analyses

The cytotoxicity and anti-inflammatory effects are presented in Table 6. When comparing the EPP-AF^®^ and EPP-AF^®^ i-CAPS, the latter exhibited superior performance in both the cytotoxicity and anti-inflammatory assays, with lower GI_50_ and IC_50_ values, respectively. However, significant toxicity was observed for the control PLP2 cells. Therefore, the potential pharmaceutical application of these extracts must be carefully evaluated, considering the balance between their cytotoxic effects on tumor cells and their toxicity towards PLP2 cells. Notably, the most promising activity was observed for EPP-AF^®^ i-CAPS, with inhibitory effects on the growth of AGS and Caco-2 cell lines at concentrations below those affecting the control PLP2 cells.

The extracts were also applied to Caco-2 cell monolayers cultivated in specific inserts to measure transport through them for hypocholesterolemic activity (Table 7). In the absorption assay of the extracts on Caco-2 cells, after 1 h incubation, the cholesterol levels in the upper compartment were significantly higher in comparison with the control assay, which is a result of the barrier caused by the adsorption of the molecules present in the extracts. In the control, the amount of cholesterol was slightly higher in the upper compartment compared to the bottom, while for the extracts the differences between compartments were significant with 72/27% for EPP-AF^®^ and 78/20% for the EPP-AF^®^ i-CAPS. Comparatively, both samples presented higher hypocholestorolemic activity, corresponding to a reduction in the release of cholesterol of 39% for the EPP-AF^®^ and even higher, 53%, for the EPP-AF^®^ i-CAPS.

#### 2.2.4. Permeability Assays on Caco-2 Cells

The Caco-2 cell monolayer permeability model has emerged as the leading in vitro tool for the assessment of potential bioavailability of drugs and other compounds in the human body, providing an excellent physical and biochemical mimic of the human intestinal epithelial membrane [24]. When cultured as a monolayer, Caco-2 cells differentiate to form tight junctions between cells to serve as a model of paracellular movement of compounds across the monolayer. Also, Caco-2 cells express all the major transporter proteins present in the human small intestine, thus allowing passive diffusion as well as active and passive transport to be investigated [25,26].

**Figure 7:**
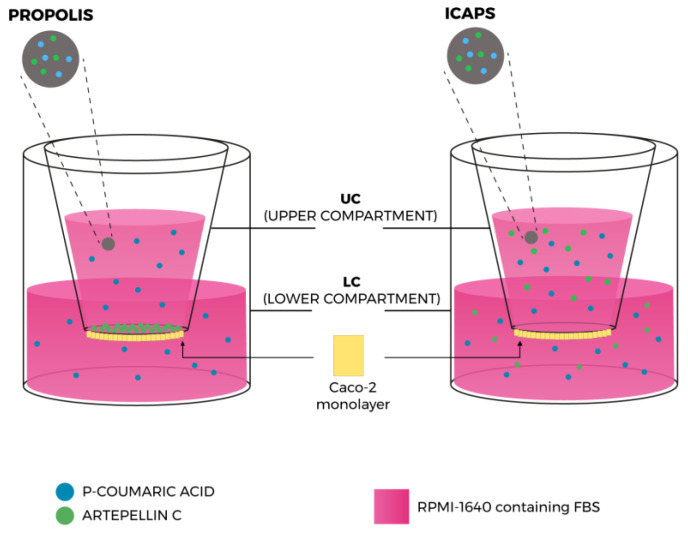
Illustration of the permeable Caco-2 monolayer transport model and results for EPP-AF^®^ (propolis) and EPP-AF^®^ i-CAPS, for the two selected biomarkers, *p*-coumaric acid and artepillin C.

**Figure 8:**
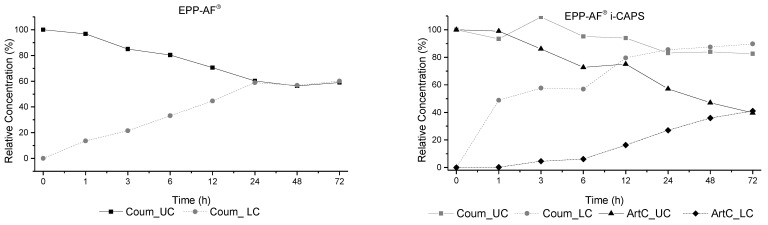
Permeability results of EPP-AF^®^ and EPP-AF^®^ i-CAPS through Caco-2 monolayer transport model (*n* = 3, mean ± SD) for the two selected biomarkers *p*-coumaric acid (Coum) and artepillin C (ArtC). UC—upper compartment; LC—lower compartment.

In our investigation, we designed a permeability study (Figure 7) in which the samples were applied to Caco-2 cell monolayers, cultivated following standard protocols, in the upper compartment. During incubation, the upper and lower compartment solutions were collected at different times (0, 1, 3, 6, 12, 24, 48, and 72 h) and the selected biomarker (*p*-coumaric acid and artepillin C) concentrations were assessed by LC/DAD/ESI-MS^n^. The results are shown in Figure 8.

In the analysis of the EPP-AF^®^ extract, due to the hydrophobic nature of artepillin C, it was only possible to quantify *p*-coumaric acid on the chromatogram at different times, although artepillin C was detected, Figure 8.

The passage of the *p*-coumaric acid through the Caco-2 cell monolayer was through normal diffusion, with no control of the release velocity from the upper compartment to the lower compartment, until it reached equilibrium at 24 h (Figure 8).

In the EPP-AF^®^ i-CAPS, both *p*-coumaric acid and artepillin C appear on the LC-DAD-ESI-MS^n^ profile. The *p*-coumaric acid showed a rapid release and permeability through the Caco-2 cell monolayer in the first hour, most probably due to its hydrophilic nature, reaching equilibrium also at 24 h. The concentration in the upper compartment did not present a proportional decrease with values from 3.20–3.01 µg/mL, until 24 h, showing that the microcapsules retain the compound. For artepillin C, the passage of the compound from the upper to the lower compartment was more proportional, but slower, reaching equilibrium only at 72 h.

## 3. Discussion

The composition of the new formulation, along with the processing system, successfully maintained the chemical profile of EPP-AF^®^ and EPP-AF^®^ i-CAPs, as confirmed by two analytical techniques, HPLC/DAD and LC/DAD/ESI-MS (Figure 1 and Table 1). To the best of our knowledge, we have demonstrated for the first time the chemical stability of a propolis extract in powder form, supporting a shelf-life of 36 months (Table 2). The degradation of key compounds, such as *p*-coumaric acid, artepillin C, and total bioactive content, remained below 15%, complying with the Brazilian regulations for phytomedicine stability [27]. Previous findings reported by Arruda et al. [28] have shown that both *p*-coumaric acid and artepillin C are highly sensitive to light and temperature. Specifically, a degradation rate of 15% was observed for *p*-coumaric acid, while artepillin C exhibited a significantly higher degradation rate of 98.1%. These degradation processes occurred under experimental conditions involving elevated temperature (40 °C) and prolonged exposure to light, spanning a duration of 30 days of analysis.

It is essential to emphasize the critical need for proper sample preparation during chemical characterization, including rupture of the microcapsules. Failing to do so may result in incomplete release of phenolic compounds and flavonoids into the quantification medium.

The spray-drying process has been widely employed for encapsulating various food ingredients, flavors, lipids, and carotenoids. It involves rapid evaporation of solvents, typically water and alcohol, resulting in the near-instantaneous encapsulation of the target compound [29,30]. Material selection plays a crucial role in optimizing operating conditions and achieving the desired efficiency. Additionally, the stability of the emulsion supplying the system becomes a critical factor when encapsulating hydrophobic compounds [29].

The photomicrographs in Figure 2 reveal that gum arabic exhibited a diverse and undefined morphology with a highly heterogeneous particle size prior to encapsulation, consistent with previous reports [23]. In contrast, the microcapsules displayed in Figure 3 demonstrated mostly spherical and well-defined shapes (Figure 3B), similar to results obtained by other researchers [23,31,32]. Busch et al. [33] also demonstrated that propolis microcapsules produced using gums exhibited more homogeneous size, fewer broken particles, spherical appearance, and more shell flexibility (avoiding fissure formation). The use of organic precursors did not affect the characteristics of the microcapsules. The average size of the microcapsules was 20 µm, and they exhibited a relatively homogeneous size distribution, except for some agglomerates, which are typical for the spray-drying process. The size and morphology were similar to those of red microcapsules obtained by Ferreira et al. [34]. However, the average size and morphology were quite different from what was obtained by Zhang et al. [13], using gum arabic and beta-cyclodextrin, but were within the range observed by Ligarda-Samanez et al. [14].

The selection of gum arabic as a carrier material proved to be a good choice due to its natural and safe characteristics. Gum arabic has demonstrated its suitability as an encapsulating agent for water-insoluble substances. Its chemical structure, consisting of neutral to slightly acidic heteropolysaccharides, including polysaccharides and glycoproteins, contributes to its functional properties [35,36]. The amphiphilic nature of gum arabic has proven effective for encapsulating and stabilizing insoluble substances, including vitamins, dyes, tocopherol, propolis, and various other natural and synthetic products [36].

The bands obtained by FT-IR for propolis samples (Figure 5) were similar to those reported by Barud et al. [37] and identified as characteristic of propolis; the bands obtained for gum arabic are also consistent with other studies [38,39]. In the vibrational spectrum of propolis microcapsules (Figure 5a), it can be observed that the bands of the extract are overlapped by the characteristic bands of the gum, indicating a change in the behavior of propolis after the emulsion and spray-drying process, demonstrating that encapsulation has occurred [40].

Based on thermogravimetry (TG/DTG) and differential scanning calorimetry (DSC) curves (Figure 5), lyophilized propolis EPP-AF^®^ has properties similar to those found in the study by Barud et al. [37]; those authors relate this behavior to simultaneous volatilization/degradation events, such as condensation of hydroxyl groups (OH-), cleavage of carbon bonds, and degradation of organic compounds. TG/DTG and DSC results presented by gum arabic were like those found in other studies [41]. And finally, the property analysis results obtained for EPP-AF^®^- i-CAPs indicated a predominance of gum arabic behavior, confirming the microencapsulation of the EPP-AF^®^ propolis extract.

The spray-drying process, with its numerous variables, such as the choice of wall materials, the proportions of materials, the ingredients to be encapsulated, and the drying conditions (e.g., inlet and outlet temperatures, flow rates), can be considered more of an art than a science. Optimization of these factors and understanding of the complex mass and heat transfer phenomena involved in microcapsule formation contribute to the intricacy of the process [29]. Achieving a high microencapsulation percentage of 93.68 ± 0.7% is a significant success. This high value, along with the morphology, FIT-IR, TG/DTG/DSC characterization curves, and release study results, demonstrate the sustained release characteristics of the microcapsules and the improved solubilization of EPP-AF^®^ in water. Different amounts (77 and 85%) of propolis were found in the microencapsulation using Capsul^®^ and gum arabic as the microencapsulation agents, respectively [31,32]. However, in another study, Nori et al. [42], using the same process, found a microencapsulation efficiency of 66–72% with soy protein isolate and pectin. Greater efficiency was found for propolis extract encapsulated by complex coacervation with alginate and gelatin, with a value close to 100% (98.77%) [43]. However, spray drying is more suitable for large-scale production; it allows for precise control over particle size, minimizes exposure to potentially damaging conditions, offers flexibility in choosing encapsulation materials, and is generally considered a cost-effective method due to its scalability, efficiency, and integration into existing production processes [29].

The choice of coating material and encapsulation method play a critical role in determining the properties and stability of microcapsules. We achieved outstanding encapsulation efficiency by employing gum arabic in conjunction with the spray-drying method. This resulted in minimal free propolis on the surface of the particles, indicating the superior physical protection of the carrier material.

The release profile of a chemical biomarker is influenced by various factors, particularly its physicochemical characteristics and its interaction with the encapsulating agent. *p*-Coumaric acid, known for its hydrophilic nature, possesses a water solubility of 18.3 mg mL^−1^ and a low partition coefficient (logP 1.79) [44]. Artepillin C has a prenylated portion that gives the molecule a lipophilic characteristic with greater affinity for cell membranes and increased biological activity [45]. The microcapsules allowed an efficient continuous release of the markers, in greater amounts and gradually following a zero-order release kinetics model, in which the materials are released as a function of time. This mathematical model shows that the microcapsule sustained release system is characteristic of products composed of controlled release matrices [46]. These findings align with previous studies [46].

The antioxidant potential for EPP-AF^®^ was superior to the results found for EPP-AF^®^ i-CAPs (Table 5). Similar data have been reported by other researchers for the antioxidant activity of encapsulated systems [23,47,48], indicating a decrease in this property or a limitation of this protocol in the evaluation of microencapsulation systems, because the solvents used are not able to disintegrate the microcapsule system, substantially compromising the results as we found in our analysis. This decrease could be attributed to degradation of phenolic compounds responsible for the antioxidant activity of the propolis extract, which may occur during the encapsulation process using spray drying [31] and may also be responsible for the small changes observed in the relative abundance within the phenolic profile (Figure 1). Additionally, the solubility in each reaction medium can be influenced by the encapsulating system employed, potentially compromising the antioxidant activity compared to the control. In a study conducted by Andrade et al. [23], the antioxidant activity of the microcapsules was evaluated after their rupture, possibly to prevent a sustained release behavior that could compromise the observed effect, as we found. Thus, it cannot be said that the microcapsules will not work in vivo but that in the in vitro model used, it was not possible to assess a benefit for the delivery system. This result may differ in an in vivo system as a function of other factors.

From the results obtained by the broth microdilution method, it was observed that the propolis microcapsules showed greater antimicrobial activity than the propolis extract used as a control (lyophilized propolis), against both Gram-positive and Gram-negative microorganisms (Table 3). This is contrary to what was observed by other authors [42,48,49,50] when comparing the antimicrobial activity of propolis extract and microcapsules produced with other wall materials. Those authors attributed their findings to the type and concentration of the wall material used, since the microcapsule wall is a physical barrier, hindering the permeation of the propolis extract into the cell and due to the possible insoluble chelation of the wall material with the flavonoids of the propolis extract, which causes a delay in the release of flavonoids [23,48]. In our study, it was possible to observe the relative advantage of the microcapsule system in this effect when both were dispersed directly in the broth dilution medium; this was due to a greater increase in the dissolution of some propolis compounds in the microcapsule system when compared with the corresponding lyophilized extract.

Although the efficaciousness of EPP-AF^®^ i-CAPs was greater than that of lyophilized EPP-AF^®^, the results found for Gram-positive microorganisms were superior to the results found for Gram negatives. This can be attributed to differences in the cell structure of Gram-positive and Gram-negative bacteria. The cell wall of Gram-positive bacteria consists of approximately 90–95% peptidoglycan, which helps the molecules penetrate cells more easily. Gram-negative bacteria have an outer membrane composed of a double layer of phospholipids that is bound to the inner membrane by lipopolysaccharides. Polysaccharides provide a barrier to penetration and are responsible for the antigenicity, toxicity, and virulence of Gram-negative bacteria, which are more resistant to natural extracts with antimicrobial activity [51].

The higher cytotoxicity revealed by the EPP-AF^®^ i-CAPS, mainly in the inhibitory effect on the growth of AGS and Caco-2 cells, when compared with the free extract is apparently due to the enhancement of the bioavailability of the contents of the microcapsules when compared with the free extract, as observed in the release studies. The rich phenolic compound composition of this type of propolis is in fact known to have a wide variety of biological functions, in addition to antioxidant activity, which are mainly related to modulation of carcinogenesis [52]. Previous studies reported Brazilian green propolis cytotoxic activity against AGP-01 gastric cancer cells, where the main compounds, artepillin C and *p*-coumaric acid, contribute to these activities [52]. Especially, artepillin C, a prenylated derivative of *p*-coumaric acid, induces apoptosis, inhibits proliferation, exerts cytotoxic effects, and inhibits autophagy [53]. The same effect was observed for anti-inflammatory activity, as the EPP-AF^®^ i-CAPS presented higher activity compared to the free extract (Table 6). Previously, immunomodulatory and anti-inflammatory activities through the inhibition of proinflammatory cytokines and increases in anti-inflammatory cytokines were described for green propolis extracts [54].

The hypocholesterolemic activity of EPP-AF^®^ and EPP-AF^®^ i-CAPS was demonstrated in the Caco-2 monolayer transport model, with both samples showing high activity, with a reduction in the release of cholesterol of 39% for the EPP-AF^®^ and even higher, 53%, for the EPP-AF^®^ i-CAPS, demonstrating enhancement of bioactivity by the microcapsules (Table 7). Previous research had already demonstrated regulation of blood lipid levels by propolis [55].

The results of the permeability assay on Caco-2 cells were similar to those of previous release studies. The passage through the cell monolayer was influenced by the microcapsules (Figure 8B). For *p*-coumaric acid, the passage was faster and more controlled, while artepillin C was detected but not quantified in the free extract (Figure 8A); the microcapsules increased the bioavailability in the aqueous medium. This behavior of artepillin C, as previously suggested, could be due to the higher affinity of the compound for the microcapsules compared to the water medium, making release slower. The Caco-2 cell monolayer permeability assay for the biomarkers indicated enhanced permeability across Caco-2 cell monolayers for the EPP-AF^®^-loaded microcapsules when compared to the free extract, with a potential increase in bioavailability.

In conclusion, the development of innovative technologies aiming to enhance the bioavailability and absorption of propolis extract, using ingredients and technology compatible with organic food supplements, is very promising. This advancement not only caters to the rising demand for functional and natural products but also opens new avenues for promoting human health and well-being [56]. By embracing these advancements, the organic food supplement industry, as well as the organic hygiene and cosmetic products industry, can meet the evolving needs and preferences of health-conscious consumers worldwide.

## 4. Materials and Methods

### 4.1. Standards and Reagents

Standard compounds, such as chlorogenic, caffeic, and *p*-coumaric acids and galangin, were acquired from Sigma Chemical Co. (St Louis, MO, USA). Kaempferol was from Extrasynthese (Genay, France) and artepillin C was from PhytoLab (L: 111674647).

Gallic acid and quercetin were supplied by ChormaDex (Irvine, Canada), with 97.6 and 99.5% purity, respectively. Phosphoric acid, sodium carbonate, and methanol (reagent grade) were supplied by Synth^®^ (São Paulo, Brazil); aluminum chloride (Cinética^®^, München, Germany), sodium tungstate (Química Moderna^®^, Recife, Brazil), phosphomolybdic acid (Nuclear^®^) were used. Sodium acetate and ferric chloride were acquired from Labsynth^®^ (Diadema, Brazil); acetic acid from Dinâmica^®^; and hydrochloric acid and ethanol (reagent grade) from Anidrol^®^ (São Paulo, Brazil). HPLC grade dimethyl sulfoxide (DMSO), 2,4,6-Tris (2-pyridyl)-s-triazine (TPTZ); 2,2-diphenyl-1-picrylhydrazyl (DPPH), and iron(II) sulfate heptahydrate were acquired from Sigma Chemical Co. (St Louis, MO, USA). Water was obtained in a Milli-Q purification system (TGI PWS, Houston, TX, USA). HPLC grade ethanol and acetonitrile were purchased from Fisher Scientific (Leicestershire, UK). Three Gram-positive microorganisms (*S. aureus*, *S. epidermidis*, and methicillin-resistant *S. aureus* (MRSA)), and three Gram-negative microorganisms (*E. coli*, *K. pneumoniae*, and *P. aeruginosa*) were acquired from Microbiologics^®^ (Saint Cloud, MN, USA). Mueller–Hinton agar broth was from Becton, Dickinson and Company (Sparks, MD, USA).

### 4.2. LC/DAD/ESI-MS^n^ Phenolic Compounds Analysis

For the analysis, both the EPP-AF^®^ and EPP-AF^®^ i-CAPS (10 mg/mL) extracts were dissolved in 2 mL of 80% ethanol/water. The solution was filtered through a 0.22 μm membrane and kept in a freezer at −20 °C, until analysis. The LC/DAD/ESI-MS^n^ analyses were performed on a Dionex Ultimate 3000 UPLC instrument (Thermo Scientific, San Jose, CA, USA) equipped with a diode-array detector and coupled to a mass detector. The column used for high-performance liquid chromatography (HPLC) was a Macherey-Nagel Nucleosil C18 (250 mm × 4 mm id; 5 mm particle diameter, end-capped) and temperature was maintained at 30 °C. The LC conditions used followed previous work [57]. A flow rate of 1 mL/min and an injection volume of 10 µL were applied. Spectral data for all peaks were accumulated in the range of 190–600 nm. The mass spectrometer was operated in the negative ion mode using a Linear Ion Trap LTQ XL mass spectrometer (Thermo Scientific, San Jose, CA, USA) equipped with an electrospray ionization (ESI) source. ESI parameters were as follows: source voltage, 5 kV; capillary voltage, −20 V; tube lens voltage, −65 V; capillary temperature, 325 °C; and sheath and auxiliary gas flow (N2) set as 50 and 10 (arbitrary units), respectively. Mass spectra were acquired at full range acquisition covering 100–1000 *m*/*z*. For the fragmentation study, a data-dependent scan was performed by deploying collision-induced dissociation (CID). The normalized collision energy of the CID cell was set at 35 (arbitrary units). Data acquisition was carried out with the Xcalibur^®^ data system (Thermo Scientific, San Jose, CA, USA). The elucidation of the phenolic compounds was achieved by comparison of their chromatographic behavior, UV spectra, and MS information to those of reference compounds. When standards were not available, the structural information was confirmed with UV data combined with MS fragmentation patterns previously reported in the literature [18,19,20]. Quantification was achieved using calibration curves for caffeic acid (0.01–0.1 mg/mL; y = 12 x 10^7^ x − 32,750; R² = 0.999), *p*-coumaric acid (0.01–0.1 mg/mL; y = 4 x 10^6^ x − 31,598; R² = 0.999), kaempferol (0.075–1.6 mg/mL; y = 9 x 10^6^ x + 248,967; R² = 0.997), and artepillin C (0.01–0.1 mg/mL; y = 9 x 10^6^ x + 864; R² = 0.999). When the standard was not available, the compound quantification was expressed in equivalent terms of the structurally closest compound. The assays were performed in duplicate and the results expressed as mg/g of extract.

### 4.3. Propolis EPP-AF^®^ and EPP-AF^®^ Microencapsulated Extracts

The standardized EPP-AF^®^ propolis extract was obtained from a blend of organic propolis obtained from suppliers in Brazil, mostly composed of green propolis raw material. Once the blend met the previously established physical–chemical and microbiological acceptance criteria, the properly ground raw material was added to a 70–80% hydroalcoholic solution, followed by percolation and centrifugation. After filtration and complete analysis of the parameters recommended by the Ministry of Agriculture [58] and the requirements of the EPP-AF^®^ patent letter, the extract was approved to be used in the other stages of the process. Propolis EPP-AF^®^ microcapsules were obtained by dispersion of an alcoholic extract of propolis in aqueous solution containing gum arabic (40:60, propolis and gum arabic). The emulsion preparation conditions, including the proportions of the dry mass of propolis and gum arabic, were previously published by Berretta et al. [7]. The ratio of propolis: gum arabic (40:60) resulted in approximately 40% propolis *w*/*w* in microcapsules (EPP-AF^®^ i-CAPs). No other additives or ingredients, besides organic propolis extract and organic gum arabic, were used. The process included dispersing gum arabic in purified water and then, with agitation, dispersing the hydroethanolic extract, resulting in an emulsion. The emulsion was dried with a spray-drying system (Labmaq MSD 1.0, Ribeirão Preto, Brazil), using the equipment conditions defined by Berretta et al. [7]. Detailed composition and process are available in Berretta et al. [7].

### 4.4. Chemical Fingerprint and Characterization

For EPP-AF^®^ extract and EPP-AF^®^ i-CAPS, 100 mg and 75 mg, respectively, of each sample were placed in 10 mL volumetric flasks, and 5 mL of HPLC grade methanol added. The samples were agitated in an ultrasound bath for 10 min. The volume was completed with ultrapure water and the solutions were filtered using a 0.45 μm filter membrane directly into a vial.

Qualitative analyses of EPP-AF^®^ and microencapsulated extracts were performed with high-performance liquid chromatography (HPLC), using a Shimadzu apparatus equipped with a CBM-20A controller, a quaternary LC-20AT pump, an array detector of SPD-M 20A diodes, and Shimadzu LC software, version 1.21 SP1. A Shimadzu Shim-Pack CLC-ODS (M) column (4.6 mm × 250 mm, particle diameter 5 mM, pore diameter 100 Å) was used. The mobile phase consisted of a gradient of methanol and water acidified with formic acid (0.1% *v*/*v*), ranging from 20 to 95%, with a run of 77 min, at a flow rate of 0.8 mL/min. The injection volume was 10 μL. The column oven was set at 40 °C. Detection was set at 275 nm, as published by Berretta et al. [5]. The samples were added to a 10 mL volumetric flask, mixed with 5 mL of methanol (HPLC grade), and were placed in an ultrasound bath for 10 min. The volume was completed with water acidified with formic acid (0.1% *v*/*v*) pH = 2.70, homogenizing carefully. It was filtered through a 0.45 µm filter membrane, directly into a 1.5 mL vial.

### 4.5. Total Flavonoid Determination

For EPP-AF^®^ extract and EPP-AF^®^ i-CAPS, 325 mg and 100 mg, respectively, of each material were transferred to 10 mL volumetric flasks. For the EPP-AF^®^ extract, 5 mL of methanol (reagent grade) was added, and for the EPP-AF^®^ i-CAPS, 5 mL of water: methanol (1:1) was added to break the microcapsules. After the samples were homogenized in an ultrasound bath, for 10 min, the flask volume was completed with the same solvent and filtered through an analytical filter paper; a 0.5 mL aliquot of each sample was transferred to a 25 mL volumetric flask and the compounds in the sample were complexed with aluminum chloride for reading in a UV–visible spectrophotometer (Shimadzu UV-1280) at a specific wavelength, as published by Funari and Ferro [59]. The reaction was kept protected from light for 30 min and reads were made at a wavelength of 425 nm. The blank solution consisted of methanol (reagent grade) and aluminum chloride. An analytical curve was prepared with dilutions of the quercetin standard at concentrations of 4.8, 5.4, 6.0, 6.6, and 7.2 µg/mL, which also underwent the complexation process with aluminum chloride, and the equation of the calibration curve was obtained. Flavonoid content was determined from this equation and calculated in mg/g as quercetin [59].

### 4.6. Total Phenolic Compounds Determination

Total phenolic compounds were determined by a colorimetric method according to Waterman and Mole [60], with modifications. For EPP-AF^®^ extract and EPP-AF^®^ i-CAPS, 460 mg and 200 mg, respectively, of each sample were transferred to 50 mL volumetric flasks. For the EPP-AF^®^ extract, 30 mL of distilled water was added, and for the EPP-AF^®^ i-CAPS, 30 mL of water: methanol (3:2) was added to break the microcapsules. After the samples were homogenized in an ultrasound bath, for 10 min, the flask volume was completed with the same solvent and filtered through an analytical filter paper. A 1.0 mL aliquot of each sample was added to Folin–Denis reagent and 35% sodium carbonate (Na_2_CO_3_), reducing phosphomolybdic–phosphotungstic acid to phenolic hydroxyl groups, producing a blue-colored complex. The reaction was protected from light for 30 min. After this period, the samples were read at a wavelength of 760 nm using a UV–visible spectrophotometer. An analytical curve was prepared with dilutions of the gallic acid standard at concentrations of 3.2, 3.6, 4.0, 4.4, and 4.8 µg/mL, which also underwent the reduction process, and the equation of the calibration curve was obtained. The content of total polyphenols was expressed in mg/g of gallic acid [60].

### 4.7. Scanning Electron Microscopy (SEM)

FEG-SEM (JEOL JMF-6700F) was used to observe the surface topography of all samples. Samples were placed on copper supports, covered with a thin layer of carbon [37].

### 4.8. X-ray Diffraction

X-ray diffractograms were obtained using a Siemens Kristalloflex diffractometer (Siemens, Knoxville, TN, USA) with a nickel filter and CuK alpha radiation between 2 theta angles from 4 to 70°, 2 s counting time, and a glass sample holder [37].

### 4.9. Fourier Transform Infrared Spectroscopy (FT-IR)

FT-IR spectra were obtained with a Perkin-Elmer spectrometer, model 2000. Samples were ground and mixed with dry KBr in known proportions and pressed into pellets [37].

### 4.10. Thermal Analysis

Thermogravimetric curves were prepared with SDT 2960 equipment from TA Instruments. The samples were heated at a constant rate of 10 °C min^−1^ from 25 to 450 °C under a nitrogen flow rate of 70 mL min^−1^ [37].

### 4.11. Microencapsulation Efficiency (ME)

Microencapsulation efficiency [32,42] was calculated by Equation (1) The phenolic compounds were determined for the solution after rupture of microcapsules to quantify the phenolic compounds released. Two-tenths (0.2) of a gram of microencapsulated extract and 2.0 mL of ethanol were mixed in a tube stirrer and centrifuged at 3000 rpm for 2 min. Then, the spectrophotometric quantification of phenolic compounds in the supernatant was made at 760 nm. Gallic acid was used as the standard.

The rupture of the microcapsules was performed with 0.2 g of microencapsulated extract and 30 mL of water: 20 mL methanol. The extract was sonicated in an ultrasound bath for 10 min, filtered through analytical paper, and the solution was analyzed for quantification of total phenolic compounds in triplicate.

(1) $$\mathrm{ME}\%=1\;-\frac{\mathrm{phenolics}\;\mathrm{on}\;\mathrm{microcapsule}\;\mathrm{surface}}{\mathrm{total}\;\mathrm{phenolics}\;\mathrm{of}\;\mathrm{microcapsule}}\times \;100$$

### 4.12. Total Antioxidant Activity by the Method of Iron Reduction (FRAP)

The assay was performed in triplicate following the methodology of Benzie and Strain [61], with some modifications. The analytical curve was prepared with ferrous sulfate heptahydrate at concentrations of 0.14, 0.29, 0.43, 0.58, and 0.72 mM. Solutions of 0.165 mg/mL of organic propolis EPP-AF^®^ extract and 0.4 mg/mL of EPP-AF^®^ i-CAPS in 70% *v*/*v* alcohol were prepared. The solutions were homogenized for 30 min with ultrasound and filtered through filter paper. Then, 70 µL of samples and curve points and 930 µL of FRAP reagent were added to the reaction medium. The reaction mixture was incubated at 37 °C in a water bath for 30 min, protected from light, and the absorbance was determined in a spectrophotometer at a wavelength of 593 nm, using the FRAP reagent at 37 °C as a blank. Antioxidant activity by FRAP was expressed in µmol Fe(II)/mg of dry mass of propolis. The *t*-test (*p* < 0.05) was applied using the statistical program GraphPad Prism—version 6.0—to compare the results of the free and microencapsulated EPP-AF^®^ propolis extracts.

### 4.13. Total Antioxidant Activity by DPPH Free Radical Scavenging

Stock solutions of 1.03 mg/mL of organic propolis EPP-AF^®^ extract and 2.5 mg/mL of microencapsulated EPP-AF^®^ were prepared in 70% *v*/*v* alcohol. The solutions were homogenized with ultrasound for 30 min and filtered with filter paper. Then, samples were diluted in 0.1 M acetate buffer, pH 5.5, at five different concentrations, respectively pipetting 40, 60, 80, 100, and 120 µL of sample to reach a final volume of 200 µL.

In the reaction medium, 400 µL of 0.1 M acetate buffer, pH 5.5, 400 µL of 96% *v*/*v* ethanol, 20 µL of sample dilutions, and 200 µL of 200 µM DPPH reagent solution were added, in that order, to 96% ethanol *v*/*v*. A negative control was also performed in which the samples were not added to the reaction medium. The blank was prepared with 400 µL of 0.1 M acetate buffer, pH 5.5, and 600 µL of 96% *v*/*v* ethanol.

The reaction medium was incubated at room temperature, protected from light for 45 min, and the absorbance of the samples was measured in a spectrophotometer at a wavelength of 517 nm. The percentage of inhibition was expressed as IC50 values. The assay was performed in triplicate [62]. The *t*-test (*p* < 0.05) was used with the statistical program GraphPad Prism—version 6.0—to compare the results of the free propolis EPP-AF^®^ and the microcapsule extracts.

### 4.14. Antimicrobial Activity by Microdilution in Broth

First, 275 mg/mL microcapsule and 113.4 mg/mL propolis extract solutions were prepared in Mueller–Hinton broth, then 200 µL of these solutions was added to the first well of a 96-well plate series and 50% serial dilutions with Mueller–Hinton broth were made until the last well of the series. Each microorganism was activated, and a suspension of 10^8^ CFU/mL was prepared in 0.85% *w*/*v* sodium chloride solution. This suspension was diluted 1/20 in Mueller–Hinton broth and 10 µL of it was added to each well. The plates were incubated at 35 ± 2 °C under aerobic conditions for 16 to 20 h. After the incubation period, the minimum bactericidal concentration (MBC) was determined by seeding 15 µL of the contents of each well at equidistant points in Petri dishes containing Mueller–Hinton agar. The plates were incubated at 35 ± 2 °C under aerobic conditions for 24 h. After the incubation period, the plates were read, observing whether there was microbial growth to determine the MBC [63]. For the comparison of propolis EPP-AF^®^ extract and i-CAPS (without the effect of the microencapsulation, i.e., both completely dissolved in the system), both samples were diluted in 40% *v*/*v* alcohol solution to break the microcapsules, and the same procedures described above were performed.

Six microorganisms were evaluated, including three Gram-positive bacteria (*S. aureus*, *S. epidermidis*, and MRSA) and three Gram-negative bacteria (*E. coli*, *K. pneumoniae*, and *P. aeruginosa*). Mueller–Hinton broth alone was utilized as the negative control, while each inoculum diluted in MHB was employed as the positive control. The experiment was carried out in triplicate for the propolis samples and in duplicate for the ethanol control.

### 4.15. Release Test

In vitro release studies were performed using Franz diffusion cells at 37 °C with cellulose membrane (10 mm MWCO 12,000–14,000) for application of EPP-AF^®^ free propolis extract (control) and EPP-AF^®^ propolis microcapsules, in the donor compartment. The receptor solution under sink conditions was constantly stirred with a magnetic bar and in a controlled temperature water bath at 37 °C. Receptor solution samples (500 μL) were collected at the following intervals: 1.5, 3, 6, 8, 24, 48, and 72 h, and the same volume of fresh receptor solution was replaced [46].

The samples were analyzed by HPLC, and the biomarkers of propolis selected for this protocol, *p*-coumaric acid and artepillin C, were detected at 275 nm, according to the methodology described in Berretta et al. [5]. As a reference, quantification of the control and the sample was used, adding 50 mg of the sample to a 10 mL volumetric flask, adding 5 mL of HPLC grade methanol, and completing the volume with 0.1% *v*/*v* formic acid. Samples were filtered through a 0.45 µm filter prior to analysis.

### 4.16. Cytotoxicity Activity

For the evaluation of the cytotoxic activity of the different extracts, the Sulforhodamine B (SRB) assay was performed on several human tumor cell lines and in a non-tumor cell line. Briefly, three human tumor cell lines were used: AGS (stomach gastric adenocarcinoma), Caco-2 (epithelial colorectal adenocarcinoma), and MCF-7 (breast adenocarcinoma). For hepatotoxicity evaluation, a cell culture was prepared from a freshly harvested porcine liver obtained from a local slaughterhouse, according to an established procedure and it was designated as PLP2 (porcine liver primary culture). The cell growth media used were RPMI-1640 containing heat-inactivated FBS (10%), glutamine (2 mM), penicillin (100 U/mL), and streptomycin (100 μg/mL); cells were incubated at 37 °C with humidified air and 5% CO_2_. To ensure detachment and deagglomeration of the cells, trypsin (proteolytic enzyme) was added to the cultures of adherent cells prior to the assays [64]. After shedding, the cells were pelleted by addition of RPMI and centrifugation (5 min, 1200 rpm), collected, and resuspended in RPMI medium. Each cell line was prepared at the appropriate density (1.0 × 10^4^ cells/well) using 96-well plates and incubated for 24 h to effect cell attachment. The extract concentrations to be tested (400–6.25 μg/mL, in DMSO/H_2_O 50:50) were added and incubated for a further 48 h. Thereafter, cold trichloroacetic acid (10%, 100 μL) was added to fix the cells and allowed to stand for 1 h at 4 °C. The plates were then washed three times with deionized water and were air-dried. SRB solution (0.1% in 1% acetic acid, 100 μL) was added and incubated at room temperature for 30 min. The plates were then washed with acetic acid (1%) to remove excess SRB and allowed to air dry. Finally, adhered SRB was solubilized by the addition of tris-HCl (10 mM, 200 μL) and the absorbance read at 540 nm in a microplate reader (Biotek Elx800). For each cell line tested, dose–response curves were obtained and the GI_50_ values, corresponding to the concentration of extract that inhibited 50% of cell growth, were calculated. For each compound, two independent experiments were performed, each one carried out in duplicate, and the results were expressed as mean values and standard deviation (SD). Ellipticin was used as a positive control [65].

### 4.17. Anti-Inflammatory Activity

For the evaluation of anti-inflammatory activity, RAW 264.7 mouse macrophages were used, following the procedure described by Moro et al. [66] and García-Lafuente et al. [67]. Cell cultures were prepared in DMEM supplemented with heat-inactivated bovine serum (10%) and L-glutamine and maintained at 37 °C with humidified air and 5% CO_2_. Cells with active growth were scraped and adjusted to an experimental density of 5 × 10^5^ cells/mL, with a dead cell ratio of less than 5%, according to the Trypan Blue exclusion test. Cells were distributed (300 μL/well) into 96-well microplates and allowed to adhere and multiply for 24 h, incubating at 37 °C and 5% CO_2_. Subsequently, they were treated with the different solutions of the extract at a final concentration of 400 to 1.56 μg/mL, reincubating for 1 h. They were then challenged with lipopolysaccharides (LPSs, 1 μg/mL, 30 μL) for 18 h. Negative controls were prepared without addition of LPSs to observe their possible effects on the basal levels of nitric oxide (NO). For the positive control, dexamethasone (50 μM) was used. The presence of nitric oxide was determined with a Griess Reagent Kit (Promega) containing sulfanilamide, N-(1-naphthyl) ethylenediamine hydrochloride (NED), and nitrated solutions. For this, the cell solution supernatant (100 μL) was transferred to a microplate along with sulfanilamide and NED solution and mixed for 5 to 10 min at room temperature. In a 96-well microplate, a reference curve for NaNO_2_ (100 μM at 1.6 μM, y = 0.0066x + 0.1349; R² = 0.9986) was prepared. The amount of nitric oxide produced was determined by measuring the absorbance at 540 nm in an ELX800 Biotek microplate reader and comparing it with the calibration curve. Finally, the concentration of extract that caused 50% inhibition of NO production (IC50, μg/mL) was determined.

### 4.18. Hypocholesterolemic Activity

The cholesterol absorption assay was carried out according to Gil-Ramírez et al. [68]. Briefly, the Caco-2 cell line was maintained in RPMI-1640 containing FBS, glutamine (2 mM), penicillin (100 U/mL), and streptomycin (100 μg/mL) and incubated at 37 °C with humidified air and 5% CO_2_. Afterwards, the cells were placed onto a 4 cm^2^ insert membrane with 0.4 μm pore size at a density of 3 × 10^4^ cells per insert. The cell plate was incubated at 37 °C in humidified atmosphere containing 5% CO_2_. The culture medium was replaced every three days and cells were allowed to differentiate for 21 days before experiments. The integrity of the cell layer was evaluated by measuring the transepithelial electrical resistance (TEER) (EVOM2; World Precision Instruments, Sarasota, FL). Only inserts with values above 400 Ω were utilized. The samples were applied to Caco-2 cell monolayers at GI_50_ concentrations (Section 4.16) in 975 µL of incomplete medium (medium without added cholesterol) in the upper compartment. After these processes, the microplate was incubated at 37 °C with 5% CO_2_ for 1 h. Thereafter, the upper solution and the solution underneath the cell monolayer were collected for cholesterol quantification by an HPLC coupled to an ultraviolet detector to evaluate the amount of cholesterol absorbed by the Caco-2 cells.

#### Cholesterol Quantification

The sample extracts were dissolved in methanol to obtain a concentration of 20 mg/mL and filtered through a 0.2 μm nylon filter for cholesterol quantification by HPLC-UV according to Heleno et al. [65]. The equipment consisted of the same Knauer system described above. Chromatographic separation was achieved with an Inertsil 100A ODS-3 reversed-phase column (4.6 × 150 mm, 5 µm, BGB Analytik AG, Boeckten, Switzerland) operating at 35 °C (7971R Grace oven).

The mobile phase was acetonitrile/methanol (70:30, *v*/*v*) at a flow rate of 1 mL min^−1^; the injection volume was 20 μL and detection was performed at 200 nm for cholesterol. Quantification was made based on calibration curves obtained from commercial standards using the internal standard method, with cholecalciferol as the internal standard. Data were analyzed using Clarity 2.4 software (DataApex).

### 4.19. Permeability Assay on Caco-2 Cells

The samples were applied to Caco-2 cell monolayers, prepared using the same procedure described in Section 4.18, in the upper compartment. After this, the microplate was incubated for 72 h. Thereafter, the upper solution and the solution underneath the cell monolayer were collected at different times (0, 1, 3, 6, 12, 24, 48, and 72 h) for quantification of the propolis biomarkers (*p*-coumaric acid and artepillin C) using LC/DAD/ESI-MS^n^, at 280 nm. 

## 5. Conclusions

We conclude that it is possible to obtain EPP-AF^®^ propolis extract microcapsules that meet the precepts of organic production and that maintain the same technological characteristics previously published with a similar encapsulation system, with a percentage of microencapsulation efficiency of 93%. For the first time, the physical–chemical results support a shelf-life of 36 months for the EPP-AF^®^ i-CAPs, considering *p*-coumaric, artepillin C, and total bioactive components. EPP-AF^®^ i-CAPs demonstrated superior antimicrobial properties when compared to lyophilized EPP-AF^®^, higher water solubility, and sustained release. The results for antitumor activities against AGS, Caco-2, and MCF-7 cells, anti-inflammatory activity, and hypocholesterolemic activity showed superiority of EPP-AF^®^ microcapsules compared to the free extract. Higher permeability was observed for compounds in propolis-loaded microcapsules. Due to cytotoxicity potential observed in porcine liver cell models, attention is needed to adjust the effective dosage for the therapeutic/functional effect. Antioxidant capacity evaluation was compromised due to the limitations of the protocols used.

## 6. Patents

Patent request for EPP-AF^®^ i-CAPS is under preparation.

## Figures and Tables

**Table 1:** Characterization of the phenolic compounds content of Brazilian green propolis obtained by LC/DAD/ESI-MS (each value is a mean ± standard deviation, *n* = 3).

Nr	RT (min)	λ_max_ (nm)	[M-H]^−^ *m/z*	MS^2^ (% Base Peak)	Proposed Compound
1	5.03	299sh, 325	353	191 (100), 179 (8), 135 (1)	5-*O*-Caffeoylquinic acid ^a,b^
2	6.93	293, 322	179	135	Caffeic acid ^a,b^
3	9.88	310	163	135	*p*-Coumaric acid
4	11.19	294sh, 325	515	353	Dicaffeoylquinic acid ^b,c^
5	12.28	294sh, 325	515	353	Dicaffeoylquinic acid (isomer) ^b,c^
6	19.22	294sh, 325	677	515	Tricaffeoylquinic acid ^b,c^
7	25.46	293	301	283 (100), 151 (29)	Dihydrokaempferide ^b,c^
8	28.51	267, 365	285	285 (100), 257 (13), 151 (20)	Kaempferol ^a,b^
9	31.69	321	247	203	5-Isoprenyl caffeic acid ^b,d^
10	37.47	315	231	187	Drupanin ^b,c^
11	39.8	310	327	283	Dihydroconiferyl *p*-coumarate ^b,c^
12	41.26	315	315	271 (100), 241 (70), 285 (59)	Cappilartimisin A ^b,c,d^
13	49.90	266, 365	299	284	Kaempferide ^b^
14	50.65	266, 365	299	284	Kaempferide (isomer) ^b^
15	53.78	316	393	349 (100), 163 (91), 199 (52)	5-Isoprenyl caffeic acid-*p*-coumaric acid ester ^b,d^
16	54.09	319	315	245 (100), 201 (30), 271 (9)	Cappilartimisin A (isomer) ^b,d^
17	61.64	314	299	255	Artepillin C
18	62.33	284	363	187	Baccharin ^b,e^

^a^ Confirmed with standard, ^b^ confirmed with MSn fragmentation, ^c^ [18], ^d^ [19], ^e^ [20]. RT = retention time.

**Table 2:** Stability results obtained using *p*-coumaric acid, artepillin C, and the total bioactive compounds as the biomarkers for the chemical characterization of organic EPP-AF^®^-loaded microcapsules (*n* = 3, mean ± standard deviation (SD), mg/g). Quantification in mg/g made at time zero (T0) and after 24 and 36 months of shelf-life (T24 and T36).

Chemical Compounds (*n* = 3)	EPP-AF^®^ i-CAPS (T0)	EPP-AF^®^ i-CAPS (T24)	EPP-AF^®^ i-CAPS (T36)	% (T24/T0)	% (T36/T0)
	Mean ± SD	Mean ± SD	Mean ± SD		
*p*-Coumaric acid	7.090 ± 0.037	6.888 ± 0.092	6.898 ± 0.023	−2.85	−2.71
Artepillin C	33.678 ± 0.993	27.693 ± 0.811 *	28.888 ± 0.350 *	−17.77	−14.22
Total bioactive compounds	93.079 ± 1.647	84.427 ± 1.812 *	85.830 ± 0.593 *	−9.30	−7.79

* Significantly different (*p* < 0.05), when compared with T0. No differences were observed between the other results.

**Table 3:** Antioxidant activity of EPP-AF^®^ and EPP-AF^®^-loaded microcapsules extracts using FRAP and DPPH methods (*n* = 3, mean ± standard deviation (SD)).

Samples	FRAP (µmol Fe^II^/mg Propolis Dry Matter) ± SD	DPPH (IC50) (µg Propolis Dry Matter/mL) ± SD
EPP-AF^®^	2.873 ± 0.045	6.500 ± 0.062
EPP-AF^®^ i-CAPS	2.654 ± 0.062 *	7.342 ± 0.058 *

* Significantly different (*p* < 0.05)—comparison of EPP-AF^®^-loaded microcapsules with the corresponding EPP-AF^®^ extract.

**Table 4:** Minimum bactericidal concentration (MBC) against *Staphylococcus aureus* and *Escherichia coli* obtained for propolis samples diluted in hydroethanolic solution 40% *v*/*v*—results expressed as propolis dry matter (mg/mL) (*n* = 3, mean ± SD).

Samples	MBC (Mean ± SD, mg/mL)	
	*S. aureus*	*E. coli*
EPP-AF^®^	3.44 ± 0.0	27.50 ± 0.0
EPP-AF^®^ i-CAPS	3.44 ± 0.0	27.50 ± 0.0
Alcohol 40% *v*/*v*	190.0 ± 0.0 *	190.0 ± 0.0 *

* Significantly different (*p* < 0.05)—comparison of alcohol 40% *v*/*v* with EPP-AF^®^ i-CAPS and EPP-AF^®^ extract.

**Table 5:** Minimum bactericidal concentration (MBC) obtained with the samples dispersed directly in the Mueller–Hinton agar—results expressed as propolis dry matter (mg/mL) (*n* = 3, mean ± SD).

Samples	MBC (Mean ± SD, mg/mL)					
	*S. aureus*	*S. aureus* MRSA	*S. epidermidis*	*E. coli*	*K. pneumoniae*	*P. aeruginosa*
EPP-AF^®^ (lyophilized)	55.0 ± 0.0	110.0 ± 0.0	55.0 ± 0.0	110.0 ± 0.0	91.67 ± 31.75	91.67 ± 31.75
EPP-AF^®^ i-CAPS	1.72 ± 0.0 *	5.73 ± 1.98 *	6.88 ± 0.0 *	55.0 ± 0.0 *	27.50 ± 0.0 *	36.67 ± 15.88 *

* Significantly different (*p* < 0.05)—comparison of EPP-AF^®^ i-CAPS and with the corresponding EPP-AF^®^ extract.

**Table 6:** Determination of cytotoxic and anti-inflammatory activities of EPP-AF^®^ and EPP-AF^®^ i-CAPS (*n* = 3, mean ± SD).

	Cytotoxic Activity (GI_50,_ µg/mL)				Anti-Inflammatory Activity (IC_50_, µg/mL)
	AGS	Caco-2	MCF-7	PLP2	RAW 264.7
EPP-AF^®^	184 ± 2 ^a^	241 ± 20 ^a^	296 ± 23 ^a^	146 ± 11 ^a^	86 ± 3 ^a^
EPP-AF^®^ i-CAPS	154 ± 1 ^b^	117 ± 1 ^b^	271 ± 25 ^a^	156 ± 4 ^a^	59 ± 0.1 ^b^
Elipticin	1.23 ± 0.03 ^c^	1.21 ± 0.02 ^c^	1.02 ± 0.02 ^b^	1.4 ± 0.1 ^b^	-
Dexamethasone	-	-	-	-	6.3 ± 0.4

GI_50_—concentration that inhibited 50% of the net cell growth; IC_50_—sample concentration providing 50% inhibition of Nitric Oxide production. Different letters in each row represent significant differences.

**Table 7:** Hypocholesterolemic activity of EPP-AF^®^ and EPP-AF^®^ i-CAPS through Caco-2 monolayer transport model (*n* = 3, mean ± SD).

		Cholesterol (µM)	Cholesterol (%)
Control	UC	26.86 ^a^	53.7 ^a^
	LC	21.82 ^C^	43.6 ^C^
EPP-AF^®^	UC	35.96 ^b^	71.9 ^b^
	LC	13.29 ^B^	26.6 ^B^
EPP-AF^®^ i-CAPS	UC	39.06 ^c^	78.1 ^c^
	LC	10.10 ^A^	20.2 ^A^

UC—upper compartment; LC—lower compartment. Different lowercase letters in each column represent significant differences between the upper compartments, while different capital letters in each column represent significant differences between the lower compartments, both of which with a significance level of 5%.

## Data Availability

Not applicable.

## Sample Availability

Samples of the compounds were adequately stored and are available from the authors.
